# On the Evaluation of a Coupled Sequential Approach for Rotorcraft Landing Simulation

**DOI:** 10.3390/s20092540

**Published:** 2020-04-29

**Authors:** Demetrio Cristiani, Luca Colombo, Wojciech Zielinski, Claudio Sbarufatti, Francesco Cadini, Michal Dziendzikowski, Marco Giglio

**Affiliations:** 1 Dipartimento di Meccanica, Politecnico di Milano, 20156 Milano, Italy; demetrioluigi.cristiani@polimi.it (D.C.); luca1.colombo@polimi.it (L.C.); francesco.cadini@polimi.it (F.C.); marco.giglio@polimi.it (M.G.); 2 Air Force Institute of Technology, Airworth, Division, 01-494 Warsaw, Poland; wojciech.zielinski@itwl.pl (W.Z.); michal.dziendzikowski@itwl.pl (M.D.)

**Keywords:** fiber Bragg gratings, landing simulation, rotorcraft, coupled sequential method, landing structural response, finite element analysis (FEA)

## Abstract

Maximum loads acting on aircraft structures generally arise when the aircraft is undergoing some form of acceleration, such as during landing. Landing, especially when considering rotorcrafts, is thus crucial in determining the operational load spectrum, and accurate predictions on the actual health/load level of the rotorcraft structure cannot be achieved unless a database comprising the structural response in various landing conditions is available. An effective means to create a structural response database relies on the modeling and simulation of the items and phenomena of concern. The structural response to rotorcraft landing is an underrated topic in the open scientific literature, and tools for the landing event simulation are lacking. In the present work, a coupled sequential simulation strategy is proposed and experimentally verified. This approach divides the complex landing problem into two separate domains, namely a dynamic domain, which is ruled by a multibody model, and a structural domain, which relies on a finite element model (FEM). The dynamic analysis is performed first, calculating a set of intermediate parameters that are provided as input to the subsequent structural analysis. Two approaches are compared, using displacements and forces at specific airframe locations, respectively, as the link between the dynamic and structural domains.

## 1. Introduction

Landing event characterization is fundamental when aircraft structural assessment has to be performed. Load spectra are inherently affected by landings, and their accurate determination cannot be pursued without considering the landing event itself. Indeed, depending on the landing severity and occurrence, the structure might be subjected to high stress levels and non-negligible performance degradation caused by material damage, especially when the landing ranks in the harsh landing regime. The definition of harsh landing is still neither clearly defined nor well-established and is generally considered as a phenomenon that occurs whenever the landing event induces abnormal operational conditions. Whether these conditions are related to the aircraft’s structural response only or also involve the passengers’ comfort is a matter of debate. Harsh landing can thus be generally located in between normal landing operations and crash events. More specifically, harsh landing is defined by the regulatory authorities in EASA Certification Specification (CS) 25 and Federal Aviation Regulations (FAR) 25 as a landing with a vertical descent velocity exceeding 3 m/s [1,2]. The same threshold is used in the Aircraft Crash Survival Design Guide (Volume 3—Aircraft Structural Crash Resistance) [3]. However, this definition is not sufficient when the landing structural response is concerned, since the vertical descent rate alone is not the unique parameter influencing the landing severity. The structural response to landing depends on multiple variables; the total aircraft weight, the mass distribution, the landing attitude (i.e., the pitch and roll angles), the weight to lift ratio, the forward and lateral landing velocities (generally null or negligible when rotorcrafts are considered), the pitch, roll and yaw rates (if non-negligible), and other parameters related to the environment, have proven to be crucial for the landing assessment.

Nowadays, the flight crew judges the classification of the landing, and eventually establishes if the landing was harsh or not [4,5]. This judgment is generally based on subjective perception, and even when it is supported by objective data, these are insufficient to assess the structural response to landing accurately. This can lead to a biased classification of the landing conditions, thus affecting the safety of the system or leading to unnecessary time and money consuming maintenance procedures. Hence the need to be fully aware of the landing structural response. Precise knowledge of the landing event structural consequences might result in considerable economic savings and increased system safety, since maintenance would be undertaken only when necessary, and based on objective data evaluation, allowing the current maintenance philosophies to evolve into potentially more cost-effective condition-based maintenance philosophies. Understanding the relationship between the landing characteristics and their structural consequences will enable not only the estimation of the actual aircraft aging, as well as an objective classification of the landing, but furthermore the potential occurrence of structural failures, thus paving the way for the implementation of health and usage monitoring systems (HUMS).

In general, any monitoring system entails the observation of a structure over time using periodical measurements, the extraction of proper features from these measurements and the analysis and interpretation of these features to determine the current state of the system, whether the focus is on its health state or its load level [6,7]. The actual state of the investigated system can often result from a statistical comparison of the current data with a database of experimental data. The latter, depending on the examined system and on the variety of circumstances it operates in, might be economically unsustainable, as well as time-consuming to be obtained. An efficient solution to this problem lies in mathematical modeling. Indeed, using numerical models and proper simulation strategies, it is possible to virtually reproduce the phenomena of concern, developing a database of the structural response based on the most influential landing features, consistently limiting the costs of an experimental campaign. The modeling and simulation of the investigated system also allow to (i) select the most suited sensor technology, (ii) predict, in the modeling space, the sensitivity of some parameters to the features of interest, (iii) design and optimize the monitoring system.

This work aims to provide an analysis method for simulating, investigating and characterizing the landing event of a medium-size rotorcraft (Mil Mi-8 helicopter), both from a dynamic and structural point of view, thus leading the way for future implementation of load monitoring (LM) and structural health monitoring (SHM) systems. Specifically, the present work addresses the landing operational range which spans between the nominal landing operations and the harsh landings. These are determined based on the structural consequences the landing has on the airframe. The present framework is enhanced by full-scale experimental tests carried out on a Mil Mi-8 helicopter, which allowed the numerical models of the examined aircraft to be evaluated and verified, proving the legitimacy of the hereafter described method.

More specifically, the landing problem field is divided into two separate domains, namely the dynamic and the structural domain. According to the pursued strategy, the dynamic domain, which relies on a multibody model of the aircraft, takes as an input the main landing characterizing features, and (i) it returns the dynamic response to the landing event; (ii) it provides the structural domain with the proper parameters to assess the landing event structural response. This strategy provides a tool that can predict the landing structural response based on some characteristic landing parameters.

The present work is structured as follows: first, the state of the art of the subject and the related topics is given, then the landing event analysis method is presented, followed by the illustration of an insight of the experimental activity. Subsequently, the modeling of the aircraft landing is examined, and the dynamic and structural domains are investigated. Finally, a conclusive section is provided.

## 2. State of the Art

The rotorcraft open literature lacks a comprehensive method able to describe and simulate the landing event from both a dynamic and structural point of view. Furthermore, general studies on the simulation strategies of unconstrained aircraft structures subjected to landing loads are lacking. Concerning rotorcraft landing simulation related topics, the open literature mainly covers two types of research lines: (i) crashworthiness related research activities, which usually consist of experimental activities (e.g., full-scale crash tests), modeling (FE models) and simulation of the crash event; (ii) research activities related to the dynamics of the rotorcraft during the landing, such as the rotorcraft-ground interaction and the development and optimization of landing gears design.

Crashworthiness related research activities date back to the pioneering work of Hugh De Haven in the 1940s [8], which provided design guidelines that are still pertinent [9,10]. Aviation crash dynamics research, besides the experimental crash assessment of already existing aircraft, is aimed at providing meaningful design guidelines to make the aircraft structures crashworthy, minimizing the harm to the passengers [11,12], as illustrated in the Aircraft Crash Survival Design Guide [3], which is considered to be a milestone in its field [9]. Apart from full-scale crash experimental tests, in recent decades, research activity has been mainly oriented towards the development of analytical/computational tools for the accurate simulation of an airframe structural response to crash loads [13,14,15]. Resources have been invested in the validation of numerical simulations and models [14,16,17], which are nowadays supported by powerful, efficient and relatively economic calculators. Virtual modeling and simulation allow us to evaluate numerous situations that are economically unfeasible with full-scale crash testing. However, full-scale experimental tests are still the unique procedure which allows the numerical models to be validated. The National Aeronautics and Space Administration (NASA) has been one of the major players in the crashworthiness related research activities. The Impact Dynamics Research Facility (IDRF) located at NASA Langley Research Center in Hampton, Virginia, witnessed decades of full-scale crash experiments (it was converted into a full-scale crash test facility for light aircraft and rotorcraft in the early 1970s) [18]. A significant example of FE code validation and simulation procedures is represented by a research project which was initiated to demonstrate the capabilities of the state-of-the-art commercial crash simulation codes in predicting the dynamic structural response of the Sikorsky ACAP (Advanced Composite Airframe Program) helicopter, during a full-scale crash test, in 1998. The objective of the crash simulation was to evaluate the capabilities of the code (MSC.Dytran) in predicting the response of a composite airframe subjected to impact loading. The numerical results were correlated with the experimental data to validate the simulation [19]. The level of agreement obtained between the experimental and analytical data ensured the numerical modeling to be an effective tool for the design and certification of crashworthy aircrafts structures. More focused activities have investigated the crashworthiness of single helicopter components or sections, such as in [17,20,21,22]. Landing gears—mainly skid landing gears—also have been included in the crashworthiness research programs, aiming at providing and optimizing effective structures for safe landings [23,24,25,26,27].

Research activities related to the dynamic response of the rotorcraft during the landing usually focus on (i) the helicopter terrain interaction, (ii) the landing gear design and optimization, whether the landing gear is wheel or skid equipped. These two topics are generally linked and usually rely on models describing the rigid dynamics of the aircraft, neglecting the deformability of structures. Concerning the landing gear related topics, in 1981 Bell Helicopters Textron published a technical report which presented the results of an investigation summarizing the landing gear criteria for helicopters [28]. The investigation was conducted in two phases: the first phase constituted a summary of a literature survey and the second phase consists of a design study of various landing gear configurations. Useful information on the landing gear characteristics identification is also present in [3,29]. Skid landing gear operations were investigated in [25], where a multibody model of a skid landing gear is presented; plastic bending deformations of structural members, damper behavior, and the characteristics of the attachments with fuselage are reproduced. Simulations in different landing conditions are carried out, and the outcomes are compared with experimental results. The investigation is performed in various attitudes and soil conditions, and the sensitivity to soil friction factor is investigated. Another work concerning a skid landing gear was published in 2007 by the NASA, describing an experimental program to assess the impact performance of a skid gear for the use on the Wasp kit-built helicopter [23]. In recent years the application of automated robotic landing gears has seen the light. For instance, in [30] a novel solution for the hard landing mitigation is proposed: the implementation of a robotic legged landing gear system, which aims at softening the hard landings by acting as a shock absorber with a relatively large stroke, thus allowing the aircraft to decelerate over a much larger distance compared with a traditional landing gear system. The investigation of such a system was explored using a multibody dynamics simulation tool. An exhaustive study on the modeling of an articulated robot landing gear was given in [31]. Concerning the dynamic landing response, a study dealing with the analysis of the helicopter-terrain interaction is carried out in [32]; this work analyzed the significant aspects related to the interaction between rotorcrafts and the terrain using a typical medium weight helicopter, with detailed wheel landing gear and full rotor dynamics, during significant maneuvers. That work aimed to investigate the interaction between the landing devices and the terrain under realistic conditions, and analyzing the effects of realistic ground loads on the rotor components. Other works specifically focused on the rotorcraft harsh landing proposed interesting landing simulation techniques [33,34], introducing on-board monitoring techniques (e.g., health monitoring systems) able to assess the structural integrity of the helicopter fuselage [33,34,35,36,37]. In [38,39] a tool for aircraft hard landing detection is suggested, being able to reveal landing gear overloads.

The present work aims at filling a void in the aircraft landing simulation open literature, proposing an effective and efficient multidomain strategy for the landing simulation and providing an objective evaluation of the proposed methods.

## 3. Method Overview

**Figure 1:**
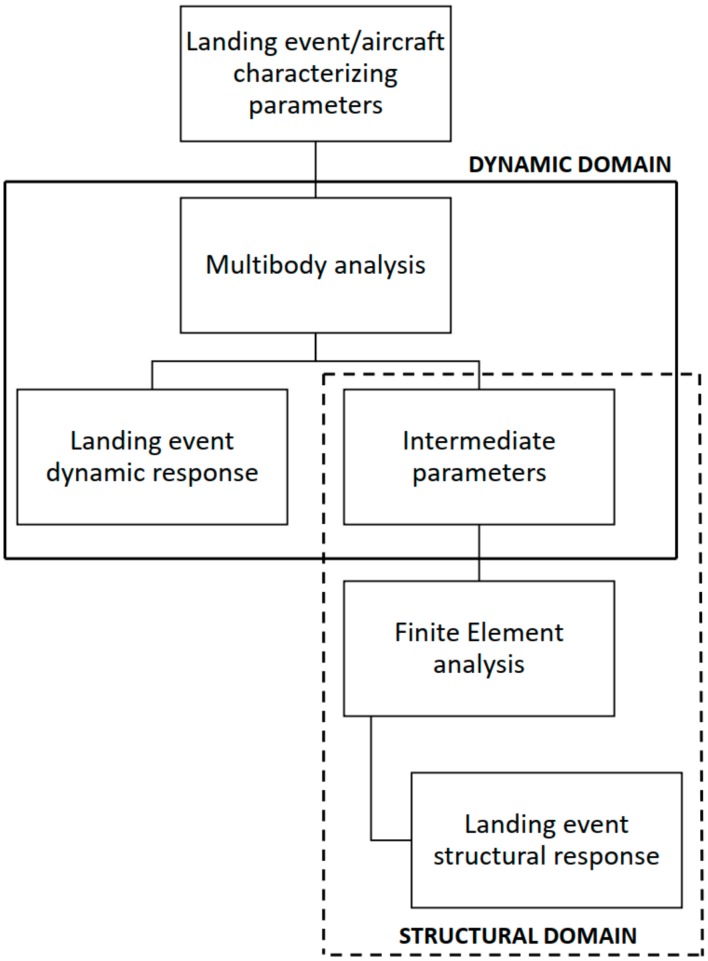
The “sequentially-coupled” method schematics.

Sequential field-coupling is the combination of analyses from different engineering disciplines that interact to solve a multidisciplinary engineering problem. When the input of one analysis belonging to one field depends on the results from another analysis belonging to another domain, the analyses are coupled. Sequential refers to the fact that the simulations are solved one after the other; the results from one analysis become the input parameters (e.g., loads and boundary conditions) for the next analysis. In the present case, the coupling is unidirectional, since data are flowing only forward, and no backpropagation nor iteration procedures take place. The pursued approach to the landing problem investigation can be thus defined as a “hybrid” or “sequentially-coupled” analysis approach, since its development involves the exploitation of two different interacting fields, resulting in a solution depending on a two-domain stepwise simulation procedure. Indeed, the complex landing problem is divided into two separate domains, which are ruled by their respective numerical models. The former domain is the dynamic one, which is governed by the multibody model of the rotorcraft. The multibody model takes as an input the landing event/aircraft characterizing features (i.e., the rotorcraft vertical touchdown velocity, mass distribution, landing attitude and weight to lift ratio), providing (i) the dynamic response to the landing and (ii) the proper parameters to feed the structural domain with, to perform the landing structural response. The latter domain is the structural one, which is ruled by a finite element model, whose loads and boundary conditions are derived directly from the dynamic analysis, as Figure 1 shows. Therefore, the described method works according to a feedforward scheme, in which the dynamic domain precedes the structural domain, providing this last domain the proper parameters to assess the landing from a structural point of view. According to the presented approach, the two domains are related in both the chronological and causal planes. Indeed, there is a clearly defined time-based relation between the two, since the dynamic domain always precedes the structural domain, besides an evident cause-effect relationship, in which the dynamic domain provides the structural domain with the parameters to perform the structural response. This strategy allows a complex multidisciplinary problem to be decoupled into two simpler monodisciplinary subproblems, making its understanding and management more effortless.

The following advantages are achieved: (i) the construction of a single overcomplex multidomain model is avoided; (ii) the two domains are fully exploited and faithfully developed, taking advantage of the specific features of each numerical model and related simulation software; (iii) detailed understanding of the dynamic response is available at a low cost, both in terms of computational power and time consumption, allowing sensitivity analyses based on the landing characterizing parameters. However, these advantages come at a cost. Indeed, the main problem associated with the present approach lies in the fact that the assumptions made in the dynamic analysis (i.e., the multibody model), which provides the structural domain (i.e., the finite element model) with the proper parameters to perform the structural response to the landing, inherently affect the structural response. Moreover, the selection of the appropriate parameters linking the two domains is not trivial, and great care has to be taken in the choice.

Concerning the intermediate parameters relating the two domains, two different approaches were followed; the former relies on a displacement control strategy, in such a way that the multibody analysis provides the finite element model with the displacements in time of some characteristic points of the airframe, namely the landing gears-fuselage attachment points. These displacements represent the boundary conditions governing the finite element analysis, which is thus time-dependent, and was performed via an implicit dynamic algorithm. The main difficulty associated with this approach lies in the fact that the multibody model is composed of rigid bodies. Therefore, enforcing these displacements in the finite element model, results in a fictitious stiffness increase of the airframe.

To overcome the issues related to the displacement control strategy, a second approach was developed, which relies on a load control strategy rather than on a displacement control strategy. Therefore, the intermediate parameters are represented by the forces exchanged by the landing gears and the fuselage. The structure being loaded and unconstrained, the inertia relief method was used [40]. In finite element analysis, the inertia relief method provides the response of an unconstrained structure subjected to constant or relatively slowly varying external loads, with static analysis computational costs. According to the inertia relief method, the unconstrained structure is assumed to be in a state of static equilibrium; indeed, an acceleration field is computed to counterbalance the applied loads. A set of translational and rotational accelerations provide distributed body forces over the structure in such a way that the sum of applied forces and the sum of moments are zero. To obtain accurate inertia relief calculation, the periods of applied loads should be much higher than the periods of rigid body modes restrained [40], so that the dynamic structural effects can be legitimately neglected. If this condition is not met, the inertia relief method might provide not meaningful results, since the stress component associated with the dynamic structural response is unaccounted for. This second approach is not exempt from issues; indeed, it does not account for the dynamic structural effects, and, unlike dynamic analyses, it is not time-dependent.

## 4. Experimental Activity

**Figure 2:**
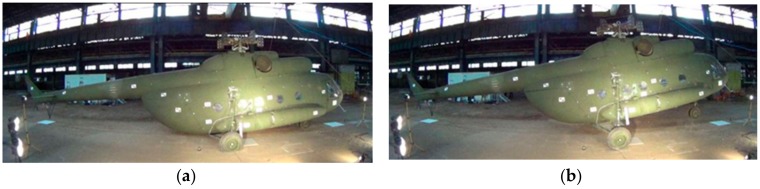
Experimental activity. Helicopter during: maximum strut compression (**a**) and front-wheel spring back (**b**).

The experimental activity consists of a set of full-scale drop tests of a medium-size Mil Mi-8 helicopter (Figure 2), in the fully-equipped full-load configuration, with gradually increasing drop heights. The Mil Mi-8 helicopter is a medium-size twin-turbine helicopter with a semi-monocoque airframe. The structure consists of an external aluminum alloy skin, stiffened with frames and stringers. The rotorcraft is provided with a wheeled landing gear structure equipped with oleo-pneumatic shock absorbers. The main landing gear (MLG) is equipped with a two-stage oleo-pneumatic absorber system, i.e., two oleo-pneumatic shock absorbers placed in series. The lower cylinder has a low-pressure low-velocity gas chamber while the upper cylinder has a high-pressure high-velocity chamber. The nose landing gear (NLG) is composed of a single oleo-pneumatic shock absorber. Since the test required the helicopter to be in a configuration as realistic as possible, the major subcomponents of the helicopter were installed on the airframe, e.g., engines, gearbox, fuel equivalent mass, etc., and additional weight representing the helicopter freight was added in the form of sandbags in the cargo compartment. The main and tail rotor blades were substituted with equivalent lumped masses. A standard gantry crane was used to perform the set of drops. The helicopter was connected to the gantry crane release mechanism employing a special lug linked to the main rotor shaft-end.

The drop height was gradually increased to raise the touchdown vertical velocity. The test was executed with the helicopter in the full weight configuration, with a total weight approximately equal to 11,000 kg.

The experimental test consisted of a set of six drops (see Table 1), with an increased drop height, starting from a 0 m height, in which the main landing gears wheels barely touched the ground and the shock absorbers were fully extended, up to a 0.78 m height, which is characterized by a vertical touchdown velocity slightly exceeding the critical one, which identifies the onset of the harsh landing regime, as stated in the introduction, according to [1,2,3]. The test configuration provided a vertical touchdown velocity direction, as for the majority of rotary-wing landings, in which the forward speed was either null or negligible. The test configuration was characterized by a zero-roll angle and a positive pitch angle equal to approximately 4 degrees, which caused the main landing gear wheels to impact the ground slightly before the nose landing gear wheels.

To acquire data from the experimental activity, a sensor network was designed and installed on the helicopter.

**Figure 3:**
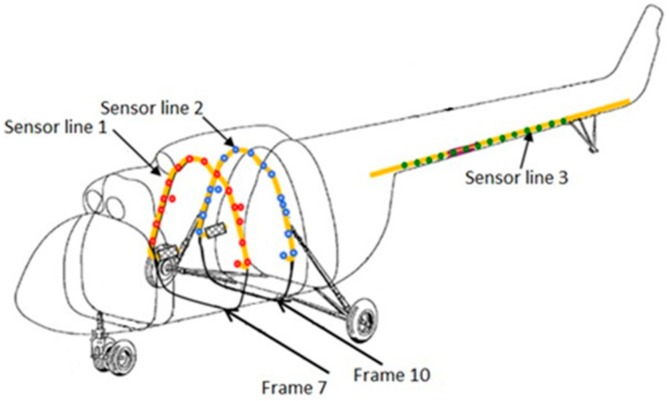
Fiber Bragg grating (FBG) sensor lines location.

The experimental measurements, besides displaying the dynamic and structural response of the aircraft to the drop experiment, also allowed to link together the drop landing parameters with (i) the parameters characterizing the dynamic response of the airframe and (ii) the stress level of the structure. Furthermore, experimental data are essential to assess and validate the numerical models of the aircraft itself and are also crucial to identify unknown or uncertainty affected parameters, as it is evidenced in the following sections. Two main categories of sensors were installed: (i) dynamic response acquisition sensors, i.e., fast camera markers, laser distance meters and shock absorbers length sensors, and (ii) structural response acquisition sensors, e.g., fiber Bragg grating (FBG) and strain gauges (SG) sensors. The former class of sensors was aimed at providing a collection of data that illustrate the dynamic behavior of the aircraft during the experimental activity. In contrast, the latter class provided data revealing the structural strains resulting from the landing event loads. Laser distance meters were fixed on the outside of the fuselage, measuring the distance between the sensor and the ground; they were located in three different points: in front of the nose landing gear, on the fuel tank in the proximity of the main landing gear, and on the tail boom extremity (Table 2). The landing event was also recorded with fast camera instrumentation (1000 frames per second); using this equipment, the displacement in time of some markers placed on the fuselage was derived. The fast camera markers were located near the main landing gear, more precisely at the fuel tank level, one forward and one rearward with respect to the main landing gear. The last camera marker was placed on the main landing gear wheel (Table 2). Shock absorbers length sensors were placed across the main landing gear shock absorbers (two shock absorbers in series configuration), measuring the stroke of the double stage suspension system (Table 2). The FBG sensors were placed in the most loaded areas, namely the frames 7 and 10 and the tail boom (Figure 3, Table 3); thus, three arrays of strain measuring sensors were available, for a total of 48 sensors, of which two were designed for temperature compensation. The FBG sensors located on the frames measured the strain along the frame circumferential direction; the ones located on the tail boom, instead, measured the axial strain with respect to the tail boom geometry. Finally, SG sensors were located side by side with some FBG sensors, to cross-check their measurements. Further experimental measurements consisted of optical scanning of the skin surface in some specific areas, performed after each drop event. Structural deformation analysis was achieved by comparing the same scanned surface morphology before and after the landing event, to detect eventual permanent (i.e., plastic) deformations [34,36].

## 5. The Dynamic Domain

The multibody model, being part of the dynamic domain, is aimed at providing the dynamic response of the rotorcraft to the landing event.

### 5.1. The Multibody Model

**Figure 4:**
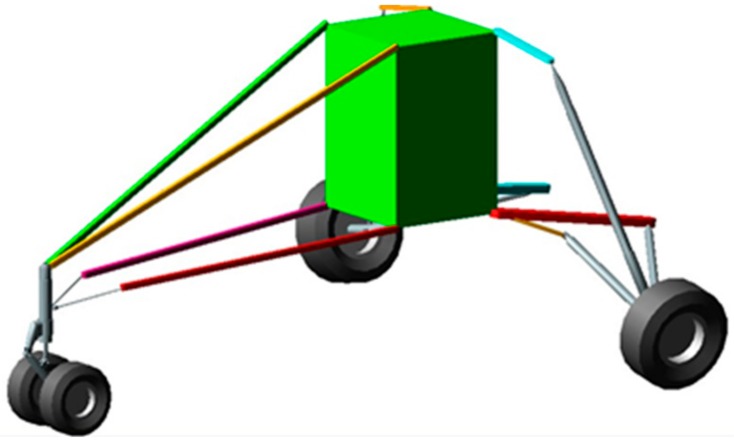
Mil Mi-8 helicopter multibody model.

The model is made up of rigid interacting bodies representing the key features of the aircraft (Figure 4). The semi-monocoque structure is described as a rigid body to which the computed inertial properties of the real fuselage structure were assigned. The landing gears were faithfully modeled, including the tire behavior. The rigid body assumption made for the fuselage structure arises from the fact that the landing gear performance, and, consequently, the general aircraft behavior, appears to be relatively unaffected by the elastic deformation of the structure [29,41], also because the landing gears fuselage attachments were located in strengthened zones, whose stiffness was thus increased. The present assumption legitimacy depends on the investigated landing regime; for instance, the more the vertical touchdown velocity is increased, the more influential the elastic structural deformation becomes. To faithfully reproduce the experimental drop test via the multibody model, this model has to devotedly reproduce the real aircraft features characterizing its dynamic behavior, i.e., its inertial properties and shock absorbers characteristics.

#### 5.1.1. The Inertial Properties

The multibody model inertial properties were extrapolated from the finite element model, which is characterized by having an accurate representation of the fuselage geometry, which was obtained via reverse engineering techniques. The semi-monocoque structure alone, including the tail boom, stabilizers, empty fuel tanks, etc., weighs approximately 1450 kg; to reach the experimental aircraft drop test weight of 11,000 kg, which is the in-service total full-load weight, a set of feature-representative masses was added to the FE model of the airframe. These masses include both the helicopter equipment and the payload. Some of the masses were modelled with solid elements, and shaped similarly to the real ones, to devotedly reproduce their inertial properties, e.g., the engines, the main rotor, the gearbox, etc., while others were modelled by one-dimensional elements, e.g., the payload, the tail rotor transmission shaft, the on-board instrumentation, etc. This process is by its very nature relatively inaccurate, and potentially introduced some errors in the modeling of the aircraft. However, the computed inertial properties are assumed to be estimated sufficiently well for the objective of this work.

#### 5.1.2. The Shock Absorbers Characteristics

**Figure 5:**
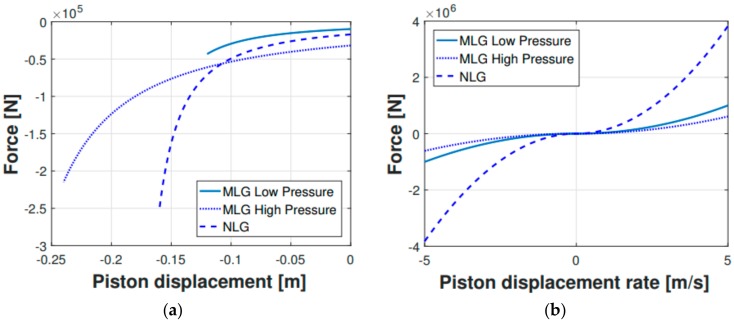
Shock absorbers elastic (**a**) and damping (**b**) characteristics.

The shock absorbers characteristics were calculated according to theoretical formulas and practical considerations [29] and were then entered in the multibody model software (MSC ADAMS). The elastic and damping characteristics were displayed as a set of force vs. displacement and force vs. rate of displacement, respectively, as illustrated in Figure 5. The forces acting on the strut are here illustrated:

(1) $$F_{S}=F_{h}+F_{a}+F_{f}$$

where: $F_{h}$ is the hydraulic force, $F_{a}$ is the pneumatic force and $F_{f}$ is the friction force. The hydraulic force in the shock strut, which is responsible for the damping characteristic, results from the pressure difference associated with the oil flow through the orifice in the oil chamber. In a landing gear, the orifice area is usually small enough in relation to the diameter of the strut so that the jet velocities and Reynolds numbers are sufficiently large that the flow is fully turbulent. As a consequence, the damping force varies as the square of the displacement rate rather than linearly. Therefore, through the Bernoulli’s principle and considering the continuity equation, the hydraulic force can be derived:

(2) $$F_{h}=\frac{\dot{s}}{\left|\dot{s}\right|}\left(p_{h}-p_{a}\right)\;A_{h}=\frac{\dot{s}}{\left|\dot{s}\right|}\;\frac{A_{h}^{3}{\dot{s}}^{2}\rho }{2{\left(\;C_{d}\;A_{n}\right)}^{2}}$$

where: $C_{d}$ is the coefficient of discharge through the orifice, $A_{n}$ is the net orifice area (assumed here to be constant), ρ is the hydraulic fluid density, $A_{h}$ is the hydraulic area, i.e., the net area on which the pressure difference is acting, and $\dot{s}$ is the displacement rate of the shock strut. The net orifice area $A_{n}$ may be either a constant value (as assumed here) or, when a metering pin is used, can vary with the strut stroke:

(3) $$\;A_{n}\left(s\right)=A_{0}-A_{p}\left(s\right)$$

where $A_{0}$ is the area of the opening in the orifice plate and $A_{p}$ is the metering pin cross section area in the orifice plate plane. Generally, the coefficient of discharge could vary because of the (i) variable net orifice area, (ii) any change in the exit and entry conditions due to variations in the length of the chambers, and (iii) because of variations in the Reynolds number of the flow. These considerations are usually neglected when dealing with constant orifice area, under the assumption of constant discharge coefficient.

The pneumatic force gives to the strut an elastic contribution, being the force dependent on the stroke. The force is determined by the initial chamber inflation pressure, the area subjected to the air pressure, and the instantaneous compression ratio. According to the polytropic law for compression of gases:

(4) $$\;P_{a}\;V_{a}^{n}=P_{a0}\;V_{a0}^{n}=constant$$

where: $P_{a}$ is the air pressure in the upper chamber of the strut, $P_{a0}$ is the air pressure in the upper chamber of the strut when completely extend, $V_{a}$ the air volume of the shock strut, $V_{a0}$ the air volume of the shock strut when wholly extended and n is the gas polytropic exponent. Since the instantaneous volume of the shock strut $V_{a}$ is equal to the difference between the initial volume $V_{a0}$ and the stroke s times the pneumatic area $A_{a}$, it is possible to write:

(5) $$P_{a}=P_{a0}{\left(\frac{V_{a0}}{V_{a0}-s\;A_{a}}\right)}^{n}$$

(6) $$\;F_{a}=P_{a}A_{2}$$

where: $F_{a}$ is the pneumatic force and $A_{2}$ is the external cross-sectional area of the inner cylinder. The gas polytropic exponent n depends on the compression rate and the rate of heat transfer from the gas to the surrounding environment. Low compression rate can be associated to isothermal compression, resulting in values of n approaching the value of 1; higher values of n, limited by the adiabatic compression value of 1.4, are typical of higher stroke rates. Here the compression was considered to be adiabatic (n=1.4), since the stroke rates were relatively high, and the heat transfer phenomena could be neglected. The force term associated to the friction was neglected. 

Since not every landing gear parameter was accurately known or easily identifiable and some simplifying assumptions were inevitably made in Equations (1)–(6), a parameter identification procedure was carried out to adjust the multibody model shock absorbers characteristics to mimic the experimental dynamic behavior. This adjustment process was performed on the 0.48 m drop, which was selected as the reference drop, since it exhibited the touch down critical velocity that identifies the onset of the harsh landing regime, thus obtaining the elastic and damping characteristics in Figure 5. Then, a comparison check was carried out for the whole experimental drop sequence, as shown in the next section.

#### 5.1.3. The Model Verification

The vertical displacement vs. time of the characteristic points listed in Table 4 is a good indicator of the whole aircraft dynamic behavior. The experimental drops measurements come from the laser distance meters and the fast camera markers. These measurements were taken as comparison parameters. The fuselage was assumed to be rigid, and the fuselage motion was considered symmetrical to the plane containing the roll and yaw axes, which is reasonable given the fact that the aircraft is almost symmetric to the roll-yaw plane and considering that the landing was initialized with a zero-roll angle. A plane roto-translation can thus approximate the motion of the aircraft in the roll-yaw axes plane.

**Figure 6:**
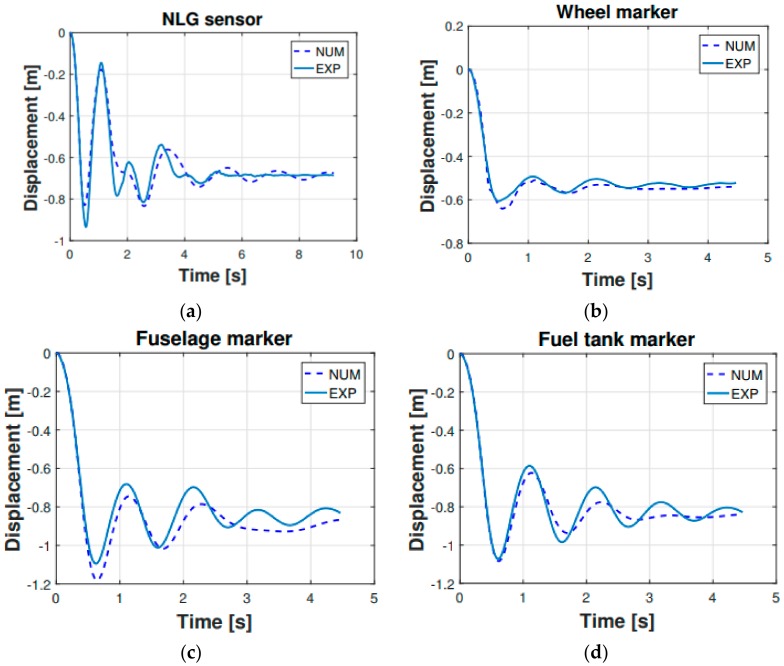
Numerical model (multibody) vs. experimental measurements of the vertical displacement for the 0.48 m drop. Displacement refers to sensors in Table 4.

**Figure 7:**
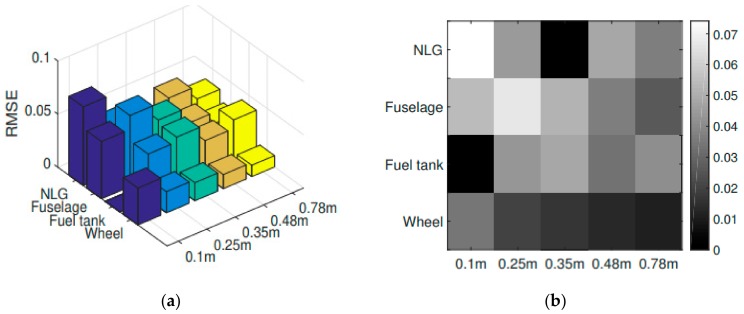
Root mean square error (RMSE) bar plot (**a**) and colormap (**b**).

Figure 6 is showing the vertical displacement vs. time of the sensors listed in Table 4, specifically for the 0.48 m drop. According to Figure 6 the multibody model satisfactorily reproduced the experimental dynamic response of the helicopter. Finally, a quantitative evaluation of the comparison is presented in Figure 7, where the root mean square error (RMSE) concerning the experimental and numerical results is shown. The RMSE was computed for each investigated sensor and including each drop height. It is useful to notice that even if the shock absorbers set-up procedure was carried out on the 0.48 m drop, the multibody model satisfactorily matched the experimentally measured behavior in the whole drop range.

**Figure 8:**
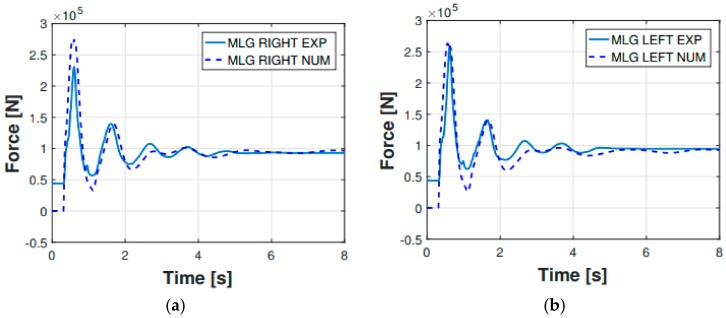
Main landing gear (MLG) shock absorbers total force for the 0.48 m drop: (**a**) right side; (**b**) left side.

**Figure 9:**
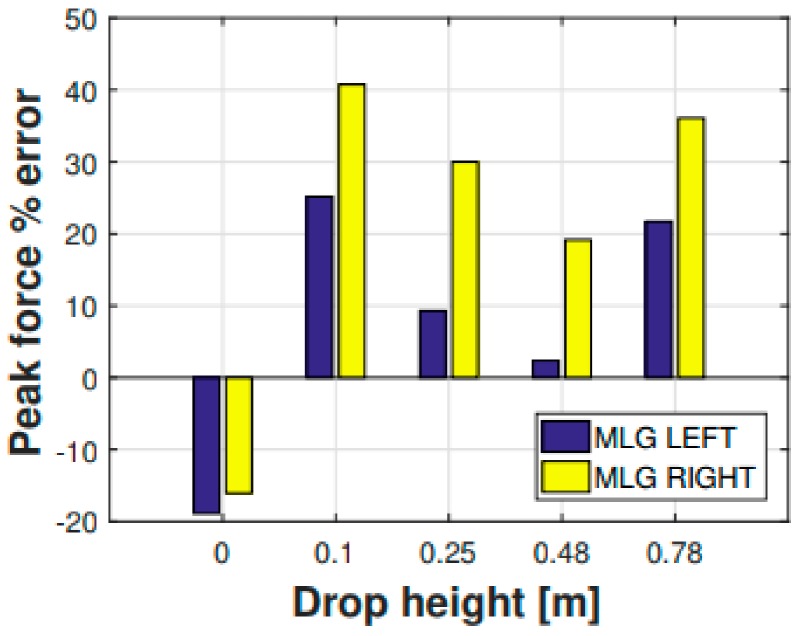
Percentage error between the experimental and numerical (multibody) MLG (right and left) peak forces for each drop height.

Apart from the displacement-based comparison ( Figure 6; Figure 7), another parameter was used as a reference to prove the legitimacy of the multibody model output parameters. This further comparison parameter is represented by the main landing gear strut loads (Figure 8 and Figure 9). The experimental forces acting on the main landing gear struts were computed via the shock absorbers stroke measurements, which were then converted—using the shock absorbers elastic and damping characteristics illustrated in Figure 5—in their related forces. In Figure 8, the comparison regarding the main landing gear forces—experimental and numerical—is shown for both the right and left side forces. The comparison refers to the 0.48 m drop. Subsequently, in Figure 9, the percentage error between the experimental and numerical peak values of the said forces was computed and illustrated.

The comparison shows that the multibody model is reproducing the real helicopter landing behavior quite accurately, both with regards to the displacements and the loads governing the structure dynamics. This entails that the intermediate parameters, whether they are the displacements or the loads acting on the fuselage, provide the finite element model with proper boundary conditions. It is finally worth noticing that further error reduction can be obtained by adopting more sophisticated models for the landing gear strut load prediction and more advanced optimization strategies, which are, however, outside the scope of the present activity.

## 6. The Structural Domain

The structural domain, which relies on finite element analysis procedures, is meant to provide the rotorcraft structural response to the landing event. A clear and comprehensive view of the structural response is fundamental when landing is assessed or classified, whether the dynamic failure criteria (e.g., fatigue criteria) is concerned or the static damage related to overload and immediately following the landing itself, enabling a reliable diagnosis of the event and providing data for the residual life prognosis.

### 6.1. The Finite Element Model

**Figure 10:**
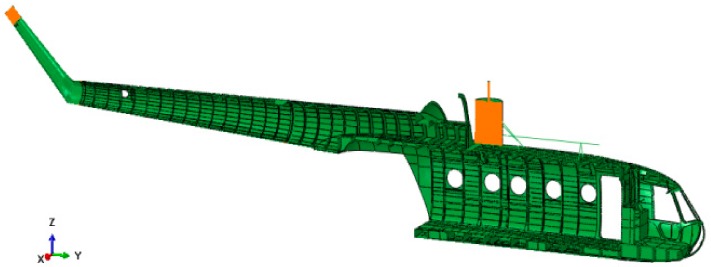
Mil Mi-8 helicopter FE model cutaway view.

The FE model was developed starting from the helicopter fuselage geometry, which was obtained employing reverse engineering techniques, and then imported in the Abaqus CAE software (Figure 10). The model consists mainly of four-node shell elements with reduced integration (S4R) and an hourglass control, characterized by a generic edge length equal to 30 mm. Some additional characteristic features which were not present in the preliminary model were added, e.g., the gearbox and its sustaining frame structure, the engines, openings in the fuselage, etc. Finally, a set of distributed masses was annexed to the fuselage to take into account the items which do not directly belong to the fuselage but were present in the experimental test, e.g., the aircraft equipment and the payload. These masses are crucial in determining both the helicopter dynamic and structural performances since they represent a large share of the total helicopter mass (87%). These additional features were modeled as lumped masses or via solid elements faithfully reproducing their key features, depending on their relevance on the global inertial properties. The fuselage is mainly made of aluminum alloy (2024 T3 alloy), whose mechanical properties are listed in Table 5.

### 6.2. The FE Model Static Verification

**Figure 11:**
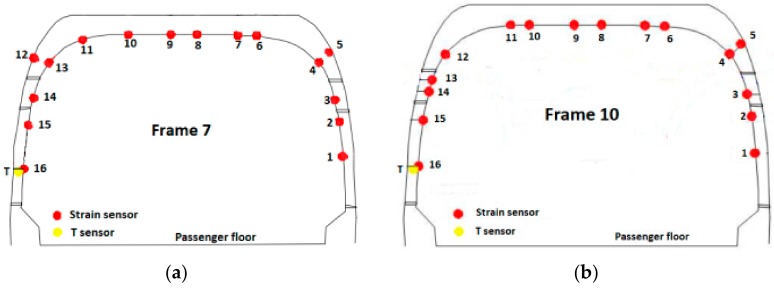

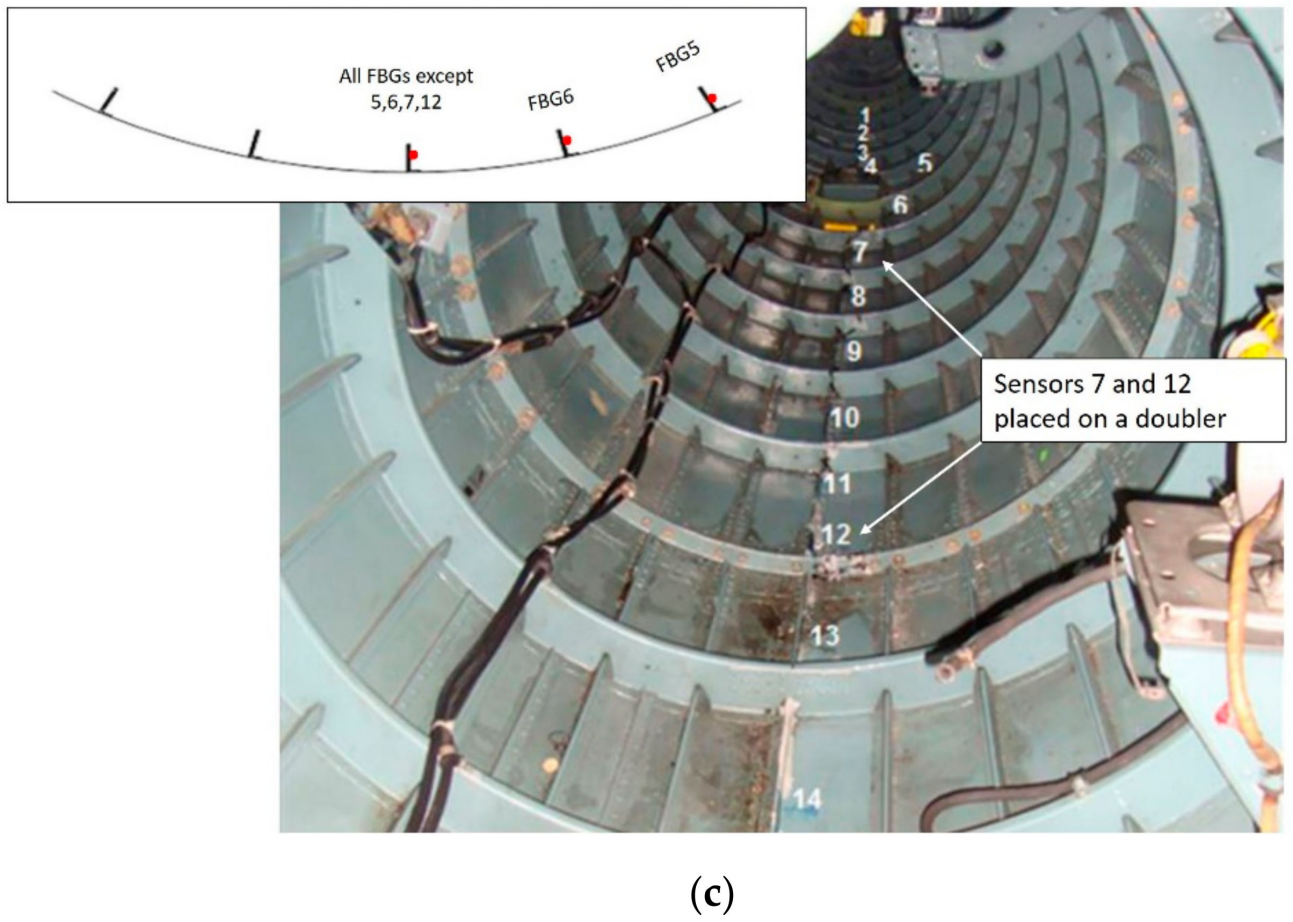
Sensor location for: (**a**) frame 7, (**b**) frame 10 and (**c**) tail boom.

**Figure 12:**
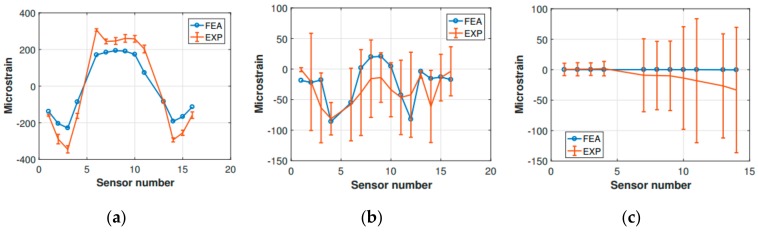
Experimental (EXP) vs. finite element analysis (FEA) strain comparison for (**a**) frame 7, (**b**) frame 10, and (**c**) tail boom.

Preliminary verification of the FE model was carried out comparing the strain measurements retrieved from the experimental activity with the corresponding numerical strains. The comparison refers to the static configuration of the helicopter resting on the ground after the landing event. Thus, the helicopter is laying on its landing gears in a static equilibrium configuration. The strain sensors (FBG sensors) location was qualitatively shown in Figure 3 and more accurately in Figure 11. The whole experimental drop data set was used, meaning that the experimental strain database includes the static “rest on-ground” configuration for each drop test, thus providing six strain samples referring to each drop experiment for each strain sensor. The relative data dispersion is shown in Figure 12.

The measured strains were calculated as perturbations with respect to the helicopter in the hung configuration (prior to the drop); as a result, the measured strain field is null in the hung configuration. The measured strain tensor can be thus formally defined as:

(7) $$\left[\varepsilon_{M}\left(t,x\right)]=[\varepsilon_{A}\left(t,x\right)]-[\varepsilon_{H}\left(t,x\right)\right]$$

The strain tensor is generally time and location dependent. As Figure 12 illustrates, frame 7 shows the best match between the FE analysis outcomes and the experimental results. In contrast, frame 10 seems to be highly sensitive to the environmental noise, having a higher data dispersion. Still, the experimental data dispersion envelope of frame 10 includes the FE strain values. The tail boom numerical strains get more accurate as the tail rotor is approached (the tail boom sensor numbering increases from the tail boom free end to the fuselage connection). In contrast, in the fuselage attachment zone the bias and the data dispersion are higher. Again, the FE strain values were included in the experimental envelope.

To objectively comment on the already presented results alone, some considerations about the FE model have to be pointed out. The finite element model suffers from having a mass distribution that is not supported by accurate data or measurements. This, in turn, affects the numerically computed structural response of the rotorcraft, since the mass distribution is crucial in determining the stress partition on the structure. Furthermore, the real aircraft structure is made up of riveted or bolted connected subcomponents; however, the FE model fuselage does not display such features. The FE model fuselage is made as a unique body, and there are no joints of any kind. Thus, the FE model exhibits an increased structural stiffness with respect to the real riveted structure, which might cause the load paths to deviate from the real ones. This increased stiffness also affects the dynamic structural behavior, since, besides having a higher stiffness, even the characteristic damping phenomena of the riveted structures are not considered. In the FE model, the modeling of substructures like the one supporting the main rotor and its gearbox, or the main landing gear strut, was proven to considerably affect the strain measurements on the frames 7 and 10. The said structures are directly in contact with frames 7 and 10, respectively, thus their influence on the investigated frames is more significant. The connection between the substructures mentioned above and the fuselage was revealed to be a central parameter, although its modeling, based on the real structure, is not straightforward.

### 6.3. The Dynamic Structural Analysis

The maximum loads acting on an aircraft structure generally occur when the aircraft is undergoing some form of acceleration, such as during landing, take-off, and other common maneuvers. From a structural point of view, the dynamic landing phase is crucial, especially when the landing is harsh, since it generates the highest loads; it is thus fundamental to focus on it. According to the present analysis approach, the loads and boundary conditions defining the structural domain are the results of the multibody analysis, while the structural strength assessment was achieved using the finite element analysis. As anticipated in Section 3, in this work, two different finite element analysis approaches were used; the former is based on a displacement control strategy, while the latter operates in the load control domain.

#### 6.3.1. The Displacement Control Strategy

The dynamic domain includes all the landing related phases in which the aircraft is not in a static equilibrium configuration. In the dynamic phase, unlike in the static one, the rotorcraft body is not constrained, and the acting loads are balanced by the inertial reaction of the structure, according to d’Alembert’s principle.

**Figure 13:**
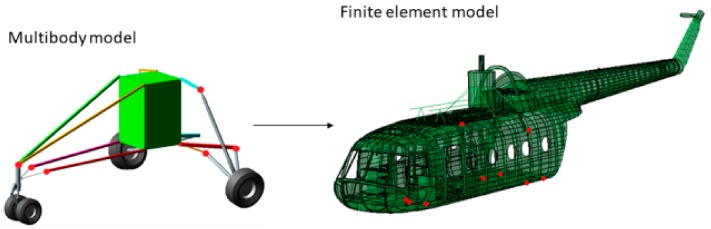
The displacement control approach schematics. Red dots represent the control points, i.e., the landing gear-fuselage attachment points.

The finite element model is the same as in the static finite element analysis; however, as the present finite element analysis was performed in the dynamic domain, a different approach was required. The basic idea behind the dynamic finite element analysis is to provide the model with the landing event loads and the boundary conditions, i.e., the displacements in some characteristic points, which result from the multibody analysis. Thus, the finite element model was provided with the displacement in time of the nine landing gears-fuselage attachment points, which are illustrated in Figure 13. The unique external load acting on the structure was the gravity load. The step was defined as implicit dynamic, neglecting the nonlinear geometric effects of large displacements, which can be ignored in the investigated scenario, especially with reference to the fuselage.

**Figure 14:**
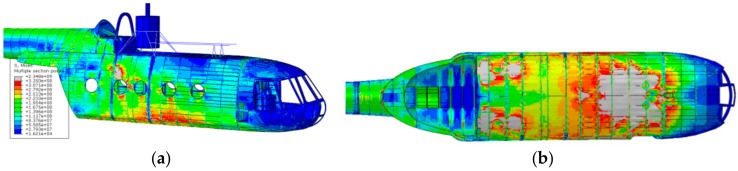
Equivalent Von Mises stress field for the 0.78 m drop. Perspective view (**a**), bottom view (**b**).

The results of the displacement control strategy are shown in Figure 14; the most critical time frame for the 0.78 m drop was shown to be 0.67 s after the release of the helicopter. This approach was affected by some relevant drawbacks:

1. The imposed motion of the landing gear-fuselage attachment points was derived from the multibody analysis. As a matter of fact, with the control point displacements derived from a rigid body model, their relative displacements were null. This, in turn, increased the finite element model structure stiffness above the actual stiffness by having a completely rigid frame connecting the control points. The abruptly increased stiffness of the structure heavily influences the loads, which travel along the stiffest pathways, which were here strongly affected by the imposed constraints. This, in turn, also affected the stress distribution.
2. Imposing the motion of the fuselage control points (and considering the rigid body assumption) made the landing gear structure geometry irrelevant. The fuselage was rigid in the multibody model, and therefore in the imposed displacements derived after the multibody analysis no evidence of the forces not parallel to the above displacements was identified.

**Figure 15:**
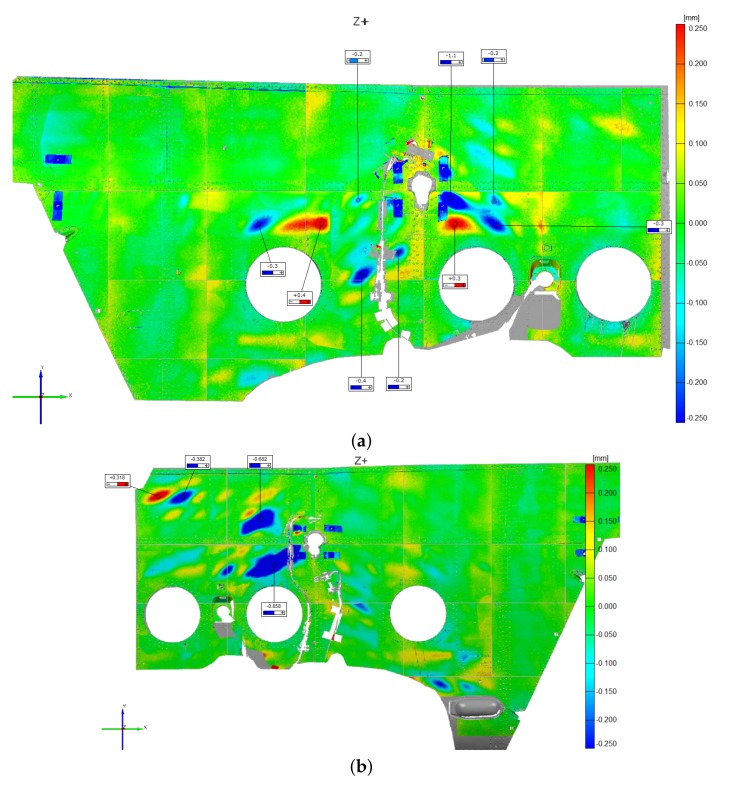

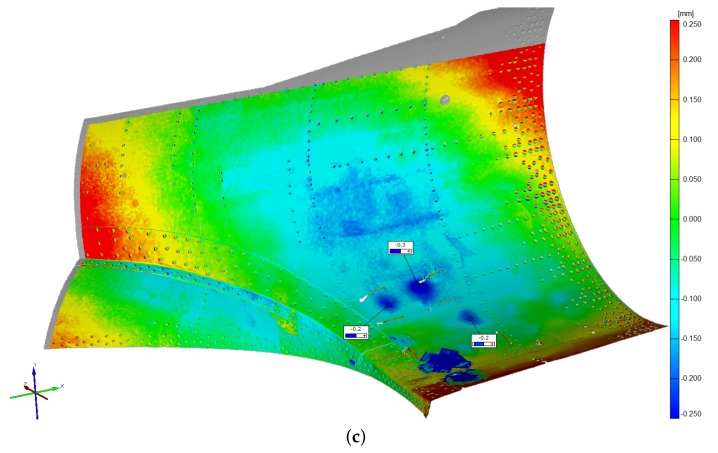
Optical scans of the fuselage skin after the 0.78 m drop. MLG attachment zone, right (**a**) and left (**b**); tail boom-fuselage connection (**c**).

These considerations were corroborated by the experimental activity findings, which derive from (i) the structure examination after each drop event and (ii) the FBG strain measurements. Concerning (i), some selected external skin regions were optically scanned after each drop attempt (an example is illustrated in Figure 15), to record detectable eventual permanent skin deformations. Structural deformation analysis was achieved by comparing the same scanned surface morphology before and after the landing event. The comparison procedure resulted in a color map of the scanned regions, which indicates the level of permanent deformation of the surface itself. The scan procedure was repeated after each drop event; thus, for each drop event, the progressive damage with respect to the previous conditions was recorded. The result of the optical scanning procedure outcomes was that only the 0.78 m drop produced any permanent structural deformation, which, however, was very localized and was not captured by the global finite element model (Figure 15); a dedicated sub modeling strategy could potentially resolve this issue. According to the specified inspection campaign, the zones prone to yielding were the following: (i) tail boom-main fuselage connection, (ii) main landing gear strut attachment area. The displacement control analysis, on the contrary, showed large yielded areas.

**Figure 16:**
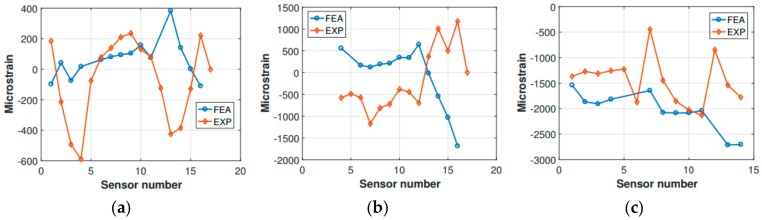
Experimental (EXP) vs. FEA strain comparison for (**a**) frame 7, (**b**) frame 10, and (**c**) tail boom.

Regarding the FBG strain measurements, these were compared with the corresponding strain values derived from the FE analysis. The comparison is illustrated in Figure 16. As expected, the comparison reflects the considerations already revealed by the qualitative observation of the FE analysis outcomes and the comparison carried out with the optical scans of the fuselage skin, highlighting the limits of the displacement control strategy. Indeed, the experimental measurements and the numerical values were inconsistent, especially when considering frame 7 and frame 10. The tail boom, instead, as already mentioned above, was less affected by the constraints imposed to the fuselage visbile in the comparison (Figure 16), which clearly showed that the tail boom FE analysis strains were much more consistent with the experimental ones.

The experimental inspection procedures suggest that there is further space for the structural model improvement with respect to the approach based on the displacement control. In the present environment, the displacement control approach was demonstrated to be inaccurate, since it largely overestimated the structural stress level, and also displayed a stress distribution which was biased due to the control points enforced rigid body motion.

Although the above considerations depict the displacement control approach as faulty, this is not necessarily always the case. Indeed, the bias of this approach with respect to the ideal structural behavior was firstly caused by the assumptions forming the basis of its implementation. Therefore, the displacement control approach might be appropriate in other contexts, since its legitimacy depends on:

1. The investigated area. If the area of interest is distant enough from the displacement control points, or if the structure is not affected by the said constraints, the bias might be tolerable, e.g., the tail boom displacement field is not affected by the main body features.
2. The multibody rigid body assumption. This hypothesis might be accurate in a wide range of scenarios. For instance, concerning the helicopter landing event, one may state that the rigid body hypothesis is less valuable with increasing landing velocity.
3. The connectivity of the displacement control points and their number, i.e., how the control region is connected to the rest of the structure. The connectivity of the control points with the rest of the structure is crucial in determining their influence. Additionally, their number is fundamental: the more there are, the higher their impact.

#### 6.3.2. The Inertia Relief Method

Aiming at providing an alternative analysis approach to the displacement control strategy, the inertia relief method (which is supported by Abaqus CAE) was applied to the landing problem. This method enables the analysis of the rotorcraft structure without the need to perform a dynamic analysis, thus permitting the examination of unsupported structures as if they were in a static equilibrium condition, as described in Section 3. Consequently, the inertia relief analysis is not time-dependent, but it investigates a single time instant. The loads are enforced to the landing gear-fuselage attachment points, i.e., the same control points used in the displacement control strategy (Figure 13). In the present analysis, the loads were selected from the multibody analysis load time history, aiming at analyzing the structure under the most critical conditions, which are likely to belong to the time window immediately following the ground impact, namely when the sum of the elastic and damping forces related to the shock absorber is at its highest. Another valuable information which has to be provided to the model is the rotorcraft attitude corresponding to the investigated time history instant. The structure attitude is fundamental, since the aircraft is inserted into the gravitational field, and the direction of the same with respect to the structure coordinate directions is essential. The rotorcraft attitude is controlled by the gravity load vector, whose components are adjusted depending on the required attitude. As stated in Section 3, to obtain accurate inertia relief analyses, the periods of the applied loads should be much greater than the periods of the rigid body modes restrained [40], so that the dynamic structural effects can be legitimately neglected. Inertia relief analysis does not account for the structure dynamic response. Depending on the structure and on the spectrum of the applied loads, the stress/strain contribution related to the structure dynamics might be relevant. Thus, the inertia relief analysis has to be applied cautiously. Here, the analysis was focused on the first instants after the ground impact (i.e., when the main landing gear loads on the structure reach their maximum value); thus, the low frequency-high displacements (i.e., high strain/stresses) contribution on the fuselage was expected not to be yet settled, while the contribution due to the elastic return subsequent the helicopter release was supposed to be irrelevant for the fuselage, while it might affect the tail boom. Ergo, the structural dynamic response does not consistently affect the overall structural response, at least as far as the fuselage is concerned; it is shown in the following section that the same might not apply for the tail boom, which is more prone to dynamic excitation.

**Figure 17:**
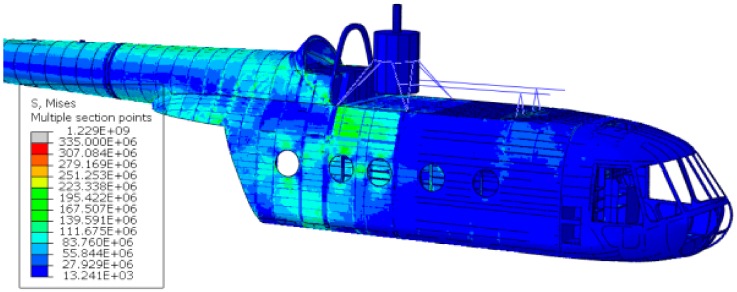
Equivalent Von Mises stress field for the 0.78 m drop.

The outcomes of the inertia relief analysis showed a completely different scenario with respect to the displacement control strategy results; firstly, the resulting stresses were far lower, and secondly, the stress distribution was rather different (Figure 17). The difference between the displacement control strategy and the inertia relief method is visible upon a comparison of Figure 17 and Figure 14. The stress magnitude overestimation is evident—the 0.78 m drop analysis conducted with the displacement control strategy displays much larger stress magnitudes than the 0.78 m drop analysis performed via the inertia relief analysis—and also the stress distribution is divergent.

As already performed for the displacement control strategy, also the inertia relief method outcomes were compared with the experimental test findings, i.e., the fuselage skin optical scans and the FBG strain measurements. First, a qualitative comparison was carried out by observing the optical scanning procedure outcomes, which are presented in Figure 15; the inertia relief analysis provided consistent results (the 0.78 m drop was considered). Indeed, it correctly detected the most stressed areas and their maximum stress magnitude was relatively adherent with the experimental findings (Figure 17). Then, to quantitatively assess the analysis, the strains derived from the inertia relief method analysis were compared to the experimental FBG strain measurements; the comparison was carried out for the 0.78 m drop, considering the time instant in which the main landing gear strut forces display their maximum value, i.e., 0.67 s after the release instant.

**Figure 18:**
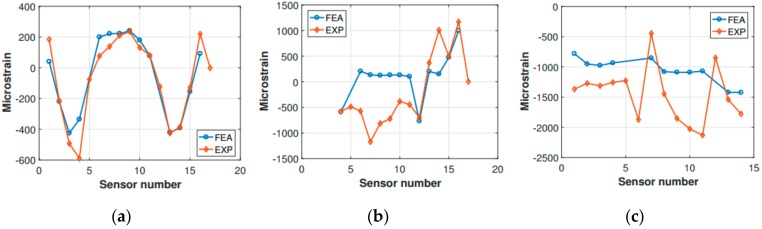
Experimental (EXP) vs. FEA strain comparison for (**a**) frame 7, (**b**) frame 10 and (**c**) tail boom.

Frames 7 and 10, and the tail boom, respectively, were compared (Figure 18). Unfortunately, not all of the experimental sensor measurements were available, since some signals were corrupted entirely by noise, and their corresponding strain measurements were discarded (Figure 18b). The comparison showed that the finite element model was entirely accurate in describing the strains which occur in frame 7, as can be observed for the static comparison (Figure 12). However, some difficulties were encountered in the characterization of frame 10. Indeed, the same kind of mismatch encountered for the static strain comparison occurred, i.e., the experimental strain values corresponding to the central section of the frame (from sensor 6 to sensor 11, Figure 18) showed a different structural behavior with respect to the finite element analysis findings. More precisely, the strain values relative to the experimental measurements were located in the negative strain half-plane, and behaved somewhat irregularly. In contrast, the finite element analysis strain values were positive and approximately around the same strain level. However, this attitude was expected, since the sensitivity of frame 10 to the external loading—the main landing gear strut was directly connected to frame 10—was already identified during the static comparison. Concerning the tail boom strain comparison, it has to be recalled that the inertia relief method is lacking the structural dynamic contribution of the tail boom structure, which behaves quite differently from the fuselage, due to its geometry and boundary conditions. Indeed, as also experimental measurements (i.e., FBG strain measurements) revealed, the tail boom undergoes oscillations as soon as the helicopter is released, since the elastic return is not negligible for the tail boom structure, which can be thought as a cantilever beam with a distributed load (its weight), plus a concentrated load on its free extremity, representing the tail rotor. Thus, when the helicopter is released from the hung position, the loads related to the gravitational field are canceled, and the elastic return of the tail boom structure causes the free vibration of the same according to the initial conditions enforced by the gravitation field. Then, when the impact on the ground takes place, the system gets excited once more, and the structure starts vibrating according to the new conditions imposed by the forces which rule the dynamic landing phase. Thus, the inertia relief method is unsuitable for assessing the tail boom strain field, and it is not generally legitimate for the simulation of systems that, according to the load spectrum, are undergoing non-negligible vibrations. In the present scenario, since the investigated instant occurred immediately after the ground impact, and the major dynamic structural contribution corresponding to the large displacements-low frequency vibration modes (i.e., large strains) was still unsettled, the inertia relief assumptions were acceptable, depending on the investigated area.

## 7. Conclusions

In the present work, a method for rotorcraft landing simulation was presented. This method fills a gap in the rotorcraft landing related open literature, providing an effective way to overcome a complex and challenging problem. The technique adopted herein allows to simulate the landing event by means of a sequentially coupled analysis strategy, which initially relies on a multibody analysis of the rotorcraft landing, and then provides the necessary input parameters to a finite element model of the rotorcraft, to assess the landing from a structural point of view.

The investigated analysis method was critically evaluated, and its legitimacy was corroborated by means of experimental data resulting from a full-scale experimental activity conducted on the examined rotorcraft. Experimental data were fundamental in the modeling phase since they enabled the numerical models of the helicopter—and the whole method also—to be objectively verified. The experimental tests also allowed the identification or adjustment of some unknown parameters, based on the experimental measurements (e.g., shock absorbers characteristics). The structural domain was investigated according to two different approaches, namely the displacement control strategy and the inertia relief method, which rely on different intermediate parameters, respectively. It was proven that the displacement control method introduced additional stiffness to the airframe (except for the tail boom), biasing the related structural response. On the other hand, this method provided a time-dependent response that accounts for the structural dynamics, and depending on the investigated area, it can provide consistent results, for instance concerning the tail boom. The inertia relief method, instead, provided consistent data with regards to the airframe, but since it does not account for the dynamic structural response, it did not accurately show the tail boom structural response, which is more prone to dynamic excitation.

The disclosed method is not relegated to rotorcrafts only, and its validity can be extended to other structures. It is essential to notice that this method, apart from being a tool for landing simulation, offers the opportunity to implement on-board monitoring strategies, allowing the creation of a comprehensive collection of structural response at a relatively low cost. This, which can be useful for assessing the structural integrity under different landing conditions or to interpret real-time signals from a network of sensors, would not be possible using experimental testing activities only, especially when considering complicated and large structures.

The present study is thus forming a solid basis for the development and implementation of on-board monitoring systems, providing a means to identify the most suitable areas for the sensor location and the most appropriate sensor technology. Future works might explore the relationship elapsing between the structural response following the landing and the quantities measured by the sensors. This would allow the monitoring system to link the measured data to a complete map of the structural response and, consequently, to the corresponding landing parameters. Vice versa, if the real-time landing parameters are known, it is possible to detect potentially harmful landings conditions, allowing the adjustment of the landing configuration.

## Figures and Tables

**Table 1:** List of drop experiments carried out.

Drop Height (m)	Freefall Touch Down Velocity (m/s)		
	Theoretical	Marker on Wheel	Error (%)
0.00	0.00	0.00	0
0.10	1.40	1.39	−0.71
0.25	2.21	2.19	−0.90
0.35	2.62	2.61	−0.38
0.48	3.07	3.01	−1.95
0.78	3.84	3.81	−0.78

**Table 2:** Dynamic response sensors.

Sensor Type	Sensor Location	Sensor Number
Laser distance meter	Nose landing gear (NLG)	1
Laser distance meter	Tail boom free-end	1
Laser distance meter	Lateral fuel tank (MLG) (right and left sides)	2
Fast camera marker	Wheel center (right side)	1
Fast camera marker	Fuselage (MLG) (right side)	1
Fast camera marker	Auxiliary fuel tank (right side)	1
Shock absorbers length sensors	MLG absorbers	4 (2 per side)

**Table 3:** Structural response sensors.

Sensor Type	Sensor Location	Sensor Number
FBG	Frame 7	17 (included T sensor)
FBG	Frame 10	17 (included T sensor)
FBG	Tail boom	14

**Table 4:** Comparison sensors.

Sensor Type	Sensor Location
Laser distance meter	Nose Landing Gear (NLG)
Fast camera marker	Wheel center
Fast camera marker	Fuselage (MLG)
Fast camera marker	Auxiliary fuel tank

**Table 5:** Fuselage material properties.

Parameter	Value
Yielding stress	335 MPa
Ultimate tensile stress	512 MPa
Young’s modulus	71,000 MPa
Poisson’s modulus	0.33
